# Fear of Movement Is Related to Trunk Stiffness in Low Back Pain

**DOI:** 10.1371/journal.pone.0067779

**Published:** 2013-06-27

**Authors:** Nicholas V. Karayannis, Rob J. E. M. Smeets, Wolbert van den Hoorn, Paul W. Hodges

**Affiliations:** 1 The University of Queensland, NHMRC Centre of Clinical Research Excellence in Spinal Pain, Injury and Health, School of Health and Rehabilitation Sciences, Brisbane, Australia; 2 Department of Rehabilitation Medicine, Research School CAPHRI, Maastricht University and Adelante Centre of Expertise in Rehabilitation and Audiology, Hoensbroek, The Netherlands; The James Cook University Hospital, United Kingdom

## Abstract

**Background:**

Psychological features have been related to trunk muscle activation patterns in low back pain (LBP). We hypothesised higher pain-related fear would relate to changes in trunk mechanical properties, such as higher trunk stiffness.

**Objectives:**

To evaluate the relationship between trunk mechanical properties and psychological features in people with recurrent LBP.

**Methods:**

The relationship between pain-related fear (Tampa Scale for Kinesiophobia, TSK; Photograph Series of Daily Activities, PHODA-SeV; Fear Avoidance Beliefs Questionnaire, FABQ; Pain Catastrophizing Scale, PCS) and trunk mechanical properties (estimated from the response of the trunk to a sudden sagittal plane forwards or backwards perturbation by unpredictable release of a load) was explored in a case-controlled study of 14 LBP participants. Regression analysis (*r* ^2^) tested the linear relationships between pain-related fear and trunk mechanical properties (trunk stiffness and damping). Mechanical properties were also compared with t-tests between groups based on stratification according to high/low scores based on median values for each psychological measure.

**Results:**

Fear of movement (TSK) was positively associated with trunk stiffness (but not damping) in response to a forward perturbation (*r^2^ = *0.33, *P* = 0.03), but not backward perturbation (*r^2^ = *0.22, *P* = 0.09). Other pain-related fear constructs (PHODA-SeV, FABQ, PCS) were not associated with trunk stiffness or damping. Trunk stiffness was greater for individuals with high kinesiophobia (TSK) for forward (*P* = 0.03) perturbations, and greater with forward perturbation for those with high fear avoidance scores (FABQ-W, *P* = 0.01).

**Conclusions:**

Fear of movement is positively (but weakly) associated with trunk stiffness. This provides preliminary support an interaction between biological and psychological features of LBP, suggesting this condition may be best understood if these domains are not considered in isolation.

## Introduction

People with low back pain (LBP) have changes in muscle activation [1], [2], trunk mechanical properties [3], [4]) and fear of pain [5], [6]. Although biological and psychological domains are often discussed in isolation, they are likely interdependent. Consistent with this view, studies have shown that adaptation in muscle activation depends on attitudes about pain [7], and compromise of the expected relaxation of the lumbar muscles at trunk flexion end range in people with LBP correlates with high fear avoidance behaviour [8]. Although it is assumed changes in trunk muscle activation relates to differences in trunk mechanical properties, the association between variation in psychological presentation and trunk mechanical behaviour has not been tested.

A relationship between psychological and mechanical features would support contemporary neurophysiology and psychology pain models, for example, the prediction of pain/threat of injury causes the body to protect the painful part in an effort to reduce pain [9], the fear-avoidance model [10], [11], and the diathesis-stress pain theory [12] (i.e., behaviours which provide short-term relief can have detrimental long-term effects if the behaviour remains unchanged [13]). We considered simultaneous investigation of biological and psychological systems could help to better understand this relationship between mechanical and behavioural domains.

Investigation of how biological and psychological features interact is of relevance because they both have the potential to influence the presentation and management of LBP. Motor control (i.e., trunk stiffness, relative tissue flexibility, preferred movement strategies) and psychological factors (i.e., emotions, cognitions, behaviours) are both likely to influence motor output and alter trunk mechanical behaviour. Depending upon the robustness of ‘motor’ and ‘psychological’ systems, it is probable the relationship is bi-directional in nature. Optimal trunk mechanical performance will vary depending upon the required task, and if a person with LBP cannot efficiently alter their mechanical response, it may have negative long-term biopsychosocial consequences (i.e., increased trunk load, reduced movement variability, reinforcement of maladaptive pain behaviour).

Several psychological features have been explored in relation to LBP, most notably dimensions related to the fear-avoidance model [14]. The three aspects of this model are fear of movement/re-injury, pain catastrophizing [15], and avoidance behaviour, all of which could relate to changes in trunk motor control. Questionnaires to assess these components have been developed [16]–[19]. There has also been a focus on distress [20], [21]. It remains unknown which psychological features, if any, are related to trunk mechanical properties. In this study we aimed to test the hypothesis that higher kinesiophobia relates to greater protection of the spine (increased trunk stiffness) by exploring the relationship between trunk mechanical properties and other aspects of the fear-avoidance model (pain catastrophizing thoughts, avoidance behaviour) as well as a component of the distress model (depression).

## Methods

### Participants

Nineteen participants with LBP (6 male, 13 female; mean body mass index (BMI) 23.6 (SD 3.8); mean age 43 (range 26–65 years)) were recruited with the objective to include those with both high and low fear of pain. Participants were included if they scored at least a 10 out of 100 on the Quebec Back Pain Disability Scale (QBPDS), reported a pain intensity of at least 1/10 at the time of participant screening (Numeric Pain Rating Scale, NPRS), and a BMI of ≤31. Exclusion criteria were a history of cancer, unexplained weight loss >4.5 kg in the past 6 months, neurologic disease, severe spinal structural deformity (e.g., >8 mm rib hump), loss of bowel or bladder control, major changes in walking balance or strength, numbness or altered sensation in the groin region, respiratory disease, hip or knee surgery or currently had a hip or knee injury, use of a walking aide, numbness in their lower extremities, or pregnancy. Participants were recruited via university and city newspaper advertisements. The Institutional Medical Ethics Committee at the University of Queensland approved the study and participants provided written informed consent.

### Procedure

#### Psychological dimensions

Participants completed questionnaires to evaluate the psychological features of their LBP. These were: Tampa Scale for Kinesiophobia (TSK), Photograph series of Daily Activities-Short electronic Version (PHODA-SeV), Fear Avoidance Beliefs Questionnaire (FABQ), Center for Epidemiological Studies-Depression Questionnaire (CES-D), Pain Catastrophizing Scale (PCS), Numeric Pain Rating Scale (NPRS), and Quebec Back Pain Disability Questionnaire (QBPDQ). .

**Figure 1:**
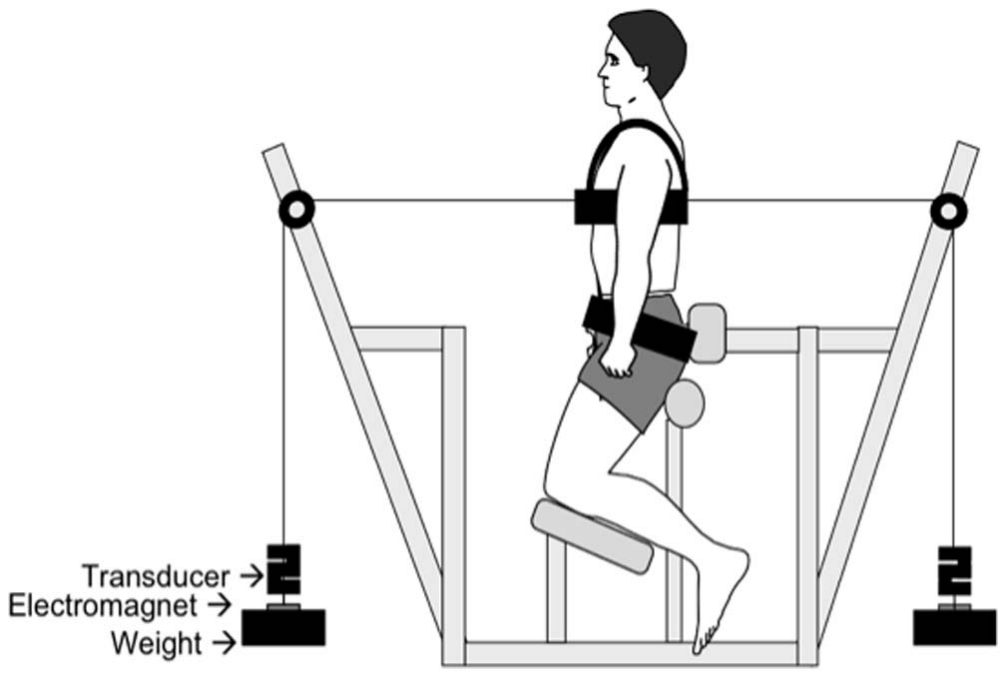
Experimental set-up. Participants sat in a semi-seated, upright posture with the pelvis fixated by a belt. The load was released from one side of the trunk by release of an electromagnet.

The TSK [17] is a 17-item measurement of fear of movement/(re) injury (1 ‘Strongly disagree’ to 4 ‘Strongly agree’) and has good reliability and validity [5]. The PHODA-SeV [19] is a valid and reliable measure of perceived harmfulness of physical activity (0 ‘Not harmful at all’ to 100 ‘Extremely harmful’) in patients with chronic LBP [19]. To gather a better comparison of the basic movement categories portrayed in the PHODA-SeV and the experimental tasks that would be performed by the participants, an additional component was added to the PHODA-SeV that involved a photograph and explanation of the experimental trunk perturbation task (see below) (Photograph of Experimental Task [PHOET]) (Figure 1). Participants rated their perceived harmfulness of participating in this task according to the same ‘harmfulness thermometer’ used in the PHODA-SeV before and after completing the test.

The PCS [18] is a 13-item questionnaire that reliably measures thoughts and feelings related to pain which suggest catastrophic thinking [22]. The FABQ [16] measure avoidant behaviour with 16 items that measure the agreement of statements related to Physical Activity (FABQ-PA) and Work (FABQ-W) affecting the participant’s LBP, and is a valid and reliable measure of fear-avoidance constructs for ‘chronic’ LBP patients [22].

The CES-D contains 20 items related to how often the person has felt depressed during the last week. It has high sensitivity (81.8%) and specificity (72.7%), and good predictive validity among ‘chronic’ pain patients for measurement of symptoms of depression [23]. The QBPDS [24] is a 20-item questionnaire related to how back pain affects a person’s daily life (0 ‘Not difficult at all’ to 5 ‘Unable to do’). The NPRS [25] (0 ‘No Pain’ to 10 ‘Worst imaginable pain’) was administered at the time of recruitment, before the experimental task, and immediately following the experimental task. Measures of pain (NPRS) and the PHOET were preformed at the start of the testing session and after completion of mechanical testing.

#### Mechanical dimensions

Mechanical properties of the trunk were evaluated from the response of the trunk to a sudden perturbation [3] (Figure 1). Participants sat in a semi-seated position with the pelvis stabilized by a belt and low-level backrest. They were instructed to sit in their normal preferred posture. A chest harness was placed over the participant’s shoulders and adjusted so that the attached cables were approximately at the trunk’s center of mass (T9). Cables were attached to equal weights (7.5% body weight) by electromagnets and passed over low-friction pulleys. A marker was placed on the cable to serve as a guide to ensure consistent posture between trials. Because front and back loads were equal, minimal muscle activity was required to hold the trunk upright. A load was randomly released from the front (x20) or back (x20) by deactivation of one electromagnet. Participants were instructed to return to their starting position after each perturbation. The dropped weight was re-attached, and successive drops followed every ∼5 s until completion of the trial (∼15 min).

Transducers (Futek Advanced Sensor Technology, Inc., Irvine, CA, USA) between the weights and trunk measured force. Force data were sampled at 200 Hz using a Power 1401 data acquisition system and Signal software (Cambridge Electronic Design, Cambridge, England). Data were exported and analyzed using Matlab (Mathworks, Natic, MA, USA).

### Data Analysis

Trunk ‘stiffness’ is the body’s ability to resist displacement and is the sum of passive (osseoligamentous system) and active (neuromuscular system) properties [26]. Trunk ‘damping’ is the body’s ability to resist velocity. Stiffness and damping were estimated using a second order linear model (Equation 1) based on the applied force and resultant trunk kinematics, from the time of weight release until maximum trunk displacement.
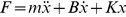
(1)


Where 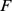 is the resultant force vector on the trunk 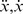 and 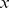 are the acceleration, velocity, and position vectors of the trunk, respectively. 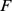 was calculated by subtracting back from front force. 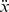 was calculated from the force transducer attached to the unreleased weight, and was numerically integrated to calculate 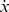 and 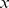. As 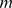
(effective mass), 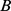 (effective damping), and 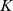 (effective stiffness) were assumed to be constant, the standard least squares procedure was used to solve the estimation. To increase the procedure’s robustness, data for the second-order linear equation were numerically integrated twice [27]. Modeled data were checked for validity by fitting a regression line between the modeled and recorded displacement data. Data were excluded from the analysis if the correlation coefficient was less than 0.97. This was identified for data from 2 participants.

To ensure normal distribution, data were transformed if Shapiro-Wilk and Shapiro-Francia test for normality was significant (*P*<0.05). The appropriate data transformation (logarithm, square root or inverse) was based on the best normal data distribution tested with skewness and kurtosis test for normality. Regression analysis was performed to test the linear relationships between trunk mechanical properties (trunk stiffness and damping) to both forward and backward perturbations and psychological factors (questionnaire scores). As an additional exploratory analysis and to provide additional support for any relationships identified in the regression analysis, data were stratified into groups with low and high scores for each psychological measure (divided by median values). The approach of using the median value or other cut-off values to divide the population based on their response to questionnaires (i.e., TSK) have been used in previous studies to describe the data [28], [29]. Mechanical variables were compared between groups with t-tests for independent samples. As data for this secondary analysis was performed in an exploratory manner for further interpretation of regression analyses, a Bonferroni correction was not used as this was considered too conservative in this hypothesis-driven context [30]. NPRS and PHOET were compared between pre- and post-test measures with t-tests for dependent measures. Significance was set at *P*<0.05. Data are presented as mean and standard deviation (SD) throughout, unless stated otherwise.

## Results

**Figure 2:**
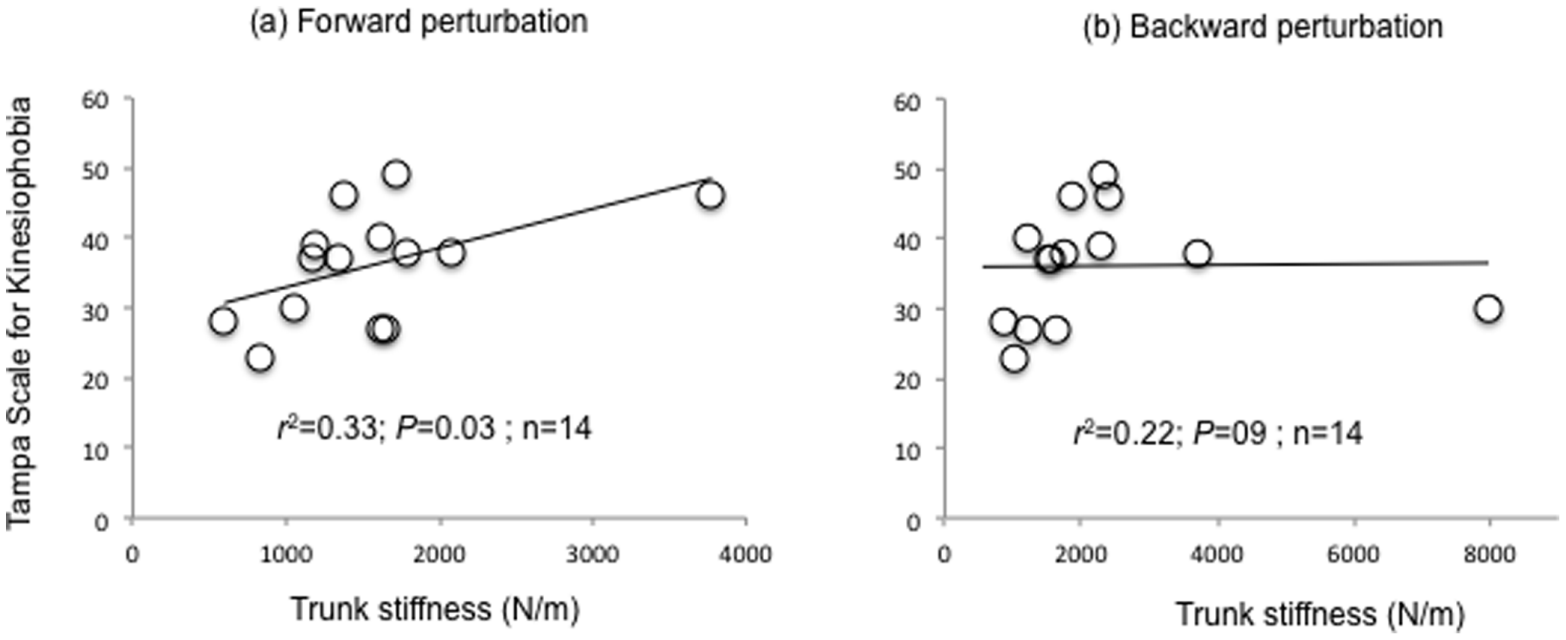
Correlation between TSK and trunk stiffness in response to (a) forward, and (b) backward perturbations.

**Figure 3:**
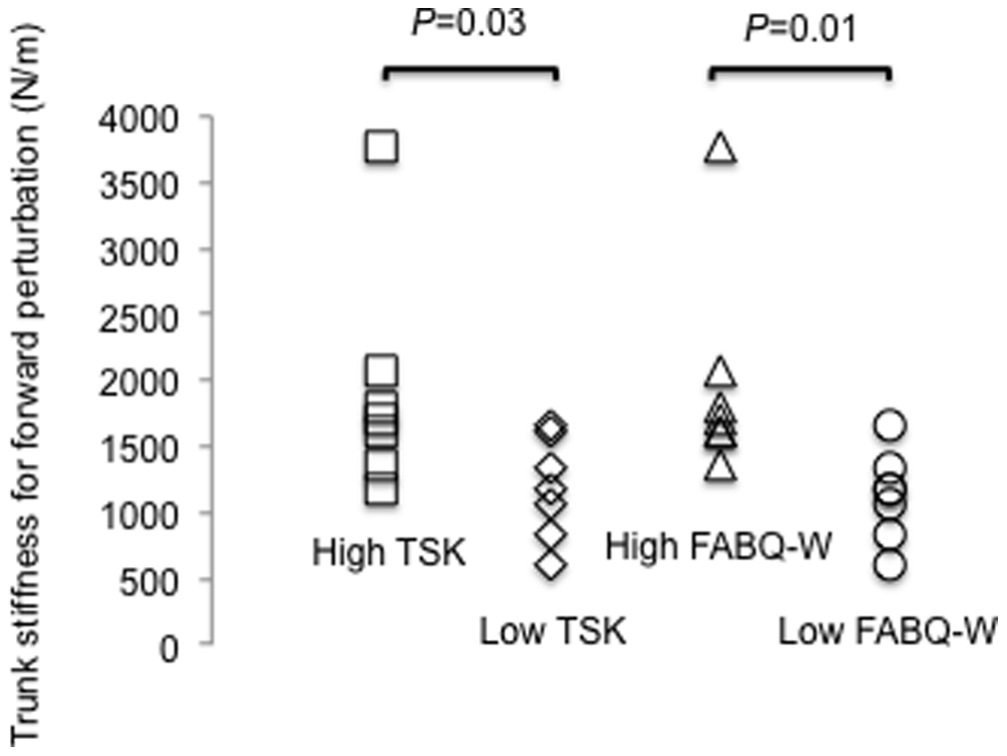
Trunk stiffness for forward perturbation for individuals with high and low kinesiophobia (TSK) and fear avoidance beliefs (FABQ). TSK = Tampa Scale for Kinesiophobia, FABQ-W = Fear Avoidance Beliefs Questionnaire-Work subscale.

Mean, range, and median values for TSK, PHODA-SeV, FABQ-W and PA subscales, PCS, CES-D, QBPDS, NPRS, age, and symptom duration are presented in Table 1. Table 2 provides comparison of normative values for participants with LBP in this study (which were lower) and 4 other studies [29], [31]–[33]. Table 3 provides a comparison of group data for trunk mechanical variables in this study and existing data [3] which used similar methods. Data for five participants were excluded from analysis due to either technical difficulties with data recording (n = 3) or failure of modelled data to adequately fit the recorded data of trunk mechanical properties (n = 2). There was a positive linear association between kinesiophobia (TSK) and trunk stiffness in response to a forward perturbation (*r^2^* = 0.33, *P*<0.03, Table 4, Figure 2). Further exploratory analysis of participants split into ‘high kinesiophobia’ and ‘low kinesiophobia’ groups based on the median TSK value (score of 38), showed higher trunk stiffness in response to forward perturbation for those with higher TSK (*P* = 0.03, Figure 3) but not backward perturbation (*P* = 0.15) (Table 5). Likewise, when participants were split into ‘high’ and ‘low’ fear avoidance groups according to the median FABQ –W and PA values, trunk stiffness was significantly greater for the “high” than “low” group during forward perturbations for the FABQ-Work subscale (*P* = 0.00). Trunk stiffness (high/low) groups for the forward perturbation were not significantly different based on scores from the FABQ-Physical Activity subscale (*P* = 0.06), nor were trunk stiffness groups significantly different during backward perturbations (Table 5). There was no significant correlation between trunk damping and kinesiophobia (Table 5) or between trunk stiffness or damping and the other psychological measures relevant to the fear-avoidance model (PHODA-SeV, PCS). The context-specific kinesiophobia measure that was related to the participant’s perceived harmfulness of the experimental task (PHOET) was not correlated with any mechanical property (Forward and backward stiffness *P* = 0.32 and 0.23, Forward and backward damping *P* = 0.45 and 0.84, respectively). Further, other measures of depression (CES-D), disability (QBPDS), pain intensity (NPRS) and age were not associated with trunk mechanical properties (Table 4). Pain was not worsened by testing (pre-test pain 2.7(2.1)/10 vs. post-test pain 2.6(2.3)/10), but the perceived harmfulness of the experimental task, as measured by the PHOET, reduced from a pre-test value of 42(22.3)/100 to post-test value of 23.2(22.6)/100).

**Table 1:** Group data for psychosocial variables.

	Mean (SD)	Range	Median
TSK	36.3/68 (7.0)	23–49	38
PHODA-SeV	35.7/100 (14.2)	9.4–56.8	38.7
FABQ-W	13.2/42 (11.4)	0.36	12
FABQ-PA	11.9/24 (5.3)	3–20	11.5
PCS	14.4/52 (8.2)	3–31	13
CES-D	11.5/60 (11.5)	4–25	9.5
QBPDS	25.7/100 (13.8)	8–57	23
NPRS (Pre-Test)	2.7/10 (2.1)	0–6.5	2
PHOET (Pre-Test)	42/100 (22.3)	10–85	40
Age	43.4 (13.2)	27–65	40
Current episode duration (weeks)	35.8 (23.3)	1–60	

TSK = Tampa Scale for Kinesiophobia, PHODA-SeV = Photographs of Daily Activities Short electronic Version, FABQ – W and PA = Fear Avoidance Beliefs Questionnaire - Work and Physical Activity subscales, PCS = Pain Catastrophizing Scale, CES-D = Center for Epidemiological Studies – Depression scale, QBPDS = Quebec Back Pain Disability Scale, NPRS = Numeric Pain Rating Scale, PHOET = Photograph of Experimental Task.

**Table 2:** Reported values for Tampa Scale for Kinesiophobia (TSK) for people with low back pain.

Study	Number of participants	TSK
Current study	19	36.3 (7.0)
Nicholas et al [31]	70	41.4 (9.0)
Smeets et al [32]	53586151	39.0 (6.5)38.7 (6.9)39.7 (7.1)37.8 (7.0)
Roelofs et al [33]	482	43.2 (8.4)
Leeuw et al [29]	85	42.0 (6.2)

**Table 3:** Current and published data for trunk mechanical properties.

		Forward Perturbation		Backward perturbation	
Study		Damping (Ns/m)	Stiffness (N/m)	Damping (Ns/m)	Stiffness (N/m)
Hodges et al [3] *					
Low back pain	Mean (SD)	17 (20)	1997 (474)	63 (39)	2035 (533)
Control	Mean (SD)	55 (37)	1641 (376)	91 (34)	1814 (471)
Current study					
Low back pain	Mean (SD)	94 (69)	1556 (753)	194 (120)	2132 (1791)
	Range	4–164	826–3775	8–325	554–7975
	Median	88	1491	218	1654

* Data for the current study relate to a perturbation induced by a removal of smaller load than that used in the study by Hodges et al. [3].

**Table 4:** Regression analysis (*r* ^2^, *P*-value) between mechanical properties and psychosocial measures.

	Forward stiffness		Backward stiffness		Forward damping		Backward damping	
	*r* ^2^	*P*-value	*r* ^2^	*P*-value	*r* ^2^	*P*-value	*r* ^2^	*P*-value
TSK	0.33	0.03*	0.22	0.09	0.02	0.60	0.24	0.07
PHODA-SeV	0.04	0.47	0.01	0.75	0.11	0.26	0.00	0.84
FABQ – W	0.18	0.12	0.01	0.69	0.01	0.69	0.12	0.22
FABQ – PA	0.21	0.12	0.04	0.50	0.05	0.47	0.03	0.60
PCS	0.21	0.10	0.01	0.77	0.02	0.66	0.10	0.23
CES – D	0.02	0.62	0.00	0.87	0.20	0.11	0.03	0.59
QBPDS	0.05	0.46	0.01	0.82	0.07	0.35	0.00	0.97
NPRS	0.00	0.99	0.01	0.82	0.07	0.36	0.00	0.33
Age	0.00	1.00	0.09	0.29	0.05	0.44	0.10	0.26

* - *P*<0.05.

Stiffness (K) = (N/m), Damping (B) = (N s/m), TSK = Tampa Scale for Kinesiophobia, PHODA-SeV = Photographs of Daily Activities Short electronic Version, FABQ - W = Fear Avoidance Beliefs Questionnaire - Work subscale, PCS = Pain Catastrophizing Scale, CES-D = Center for Epidemiological Studies – Depression scale, QBPDS = Quebec Back Pain Disability Scale, NPRS = Numeric Pain Rating Scale.

**Table 5:** *P*-values (independent t-tests) for comparison of mechanical properties between groups stratified by median value of psychosocial variables.

	Forward stiffness	Backward stiffness	Forward damping	Backward damping
High TSK (>38)	**0.03** *	0.15	0.57	0.15
High FABQ-W (>12)	**0.01** *	0.49	0.29	0.49
High FABQ-PA (>11.5)	0.06	0.21	0.80	0.21
High PCS (>13)	0.22	0.84	0.65	0.84
High PHODA-SeV (>38.7)	0.22	0.72	0.21	0.72

* = *P*<0.05.

Stiffness (K) = (N/m), Damping (B) = (N s/m). TSK = Tampa Scale for Kinesiophobia, FABQ-W & PA = Fear Avoidance Beliefs Questionnaire-Work & Physical Activity subscales, PCS = Pain Catastrophizing Scale, PHODA-SeV = Photographs of Daily Activity – Short electronic Version.

## Discussion and Conclusion

This study provides partial support for the hypothesis that psychological aspects (i.e., kinesiophobia) are not independent from the biological presentation (i.e., trunk mechanical properties) of LBP. Consistent with our hypothesis, higher measures of kinesiophobia (TSK) were associated with higher measures of trunk stiffness in response to a forward perturbation. However, trunk damping did not correlate with psychological measures. When data were further probed by stratification into groups with higher and lower scores on measures of psychological features, those with high measures of kinesiophobia (TSK) and fear avoidance beliefs (FABQ) had greater trunk stiffness in response to forward perturbations. The observations of this study imply that neither biology nor psychology should be considered in isolation for investigation or management of this multidimensional disorder.

Despite the relationship between high trunk stiffness and kinesiophobia, trunk mechanical properties were not associated with pain-related fear measures, such as perceived harmfulness (PHODA-SeV, PHOET) or pain catastrophizing (PCS). Trunk mechanical properties were also not associated with measures of disability (QBPDS) or depression (CES-D). Catastrophizing is proposed to initiate the fear-avoidance event cycle, and disability and depression are identified as consequences of elevated pain-related fear and avoidance. One interpretation of the present data is that biomechanical manifestations of pain (i.e., elevated trunk stiffness and increased superficial trunk muscle activity which is likely to contribute to the increased stiffness) are most closely associated at the pain-related fear stage of the fear-avoidance model, rather than its hypothesised precursor, catastrophization.

The basis for the significant association between mechanical properties and kinesiophobia, but not the preceding component of catastrophizing or resulting disability and depression is unclear. One possible explanation is that the proposed steps of the fear avoidance model are non-linear, and there are other ways the human system responds to catastrophizing and disability, which are not represented or manifested through these mechanical behaviors. Pain intensity and/or symptom duration could also play a role in reported fear-avoidance and distress levels, and it is important to acknowledge the relatively low pain intensity values (2.7/10 (2.1)), and persistent and recurrent symptom duration (9 months) of the participants in this study.

The lack of significant relationship between biological properties and the PHODA-SeV, PHOET, and PCS is perhaps reflective of the context or interpretation of the questionnaires. A plausible explanation for the correlation between TSK and trunk mechanical properties in the absence of relationship with the other fear-avoidance related questionnaires might be explained by wider consideration of psychological variables in this measure. The TSK contains items pertaining to a wide spectrum of beliefs (e.g., pain will increase or re-injury will occur if they increased their physical activity or exercise level; something is dangerously wrong with their LBP; they are at a greater risk of injuring themselves; pain equates to injury or danger; and they are being delegitimized). In contrast, FABQ items attempt to gain insight on: the heaviness or monotonous behaviour of their work, normal work ability, return to work expectancy, beliefs related to pain serving as an indicator that they should stop their activity, the belief that feeling pain serves as an accurate measure that something is dangerously wrong. Further, the PHODA-SeV (and PHOET) are merely asking the participant to consider which activities they consider harmful or damaging to their back and the PCS primarily contains questions related to catastrophizing/perseverating thoughts (e.g., “I keep thinking about how much it hurts”) and questions related to fear, worry, and anxiety. It could also be that the psychometric variables of the TSK are more sensitive to the particular measures of motor control included here.

### Clinical Relevance

An interpretation of the results is that kinesiophobia is more closely associated with trunk mechanical properties than other psychological factors (fear avoidance beliefs, pain catastrophizing thoughts, and depression). This finding highlights the interaction between kinesiophobia and trunk control as a potential target for future work addressing questions of causality and design of interventions.

Integration of a biopsychosocial perspective into the practice of pain management is well accepted as the gold standard of care, but disparity could arise if clinicians choose to focus effort on either a biomedical or psychological approach in isolation. The potentially false rationale for incorporating this dualistic philosophy could stem from a misleading judgement that either the mechanical factors or psychological factors are a stronger mediator of pain and/or pain behaviour, and hence, only focus the intervention along one of these domains. Another commonly held perspective is that biomechanical intervention should be augmented with psychological treatments only in cases where patients are considered to have a higher risk of involvement of psychological factors in their presentation, but comprehensive identification of those individuals at higher risk remains a challenge. Results of this study can be interpreted to suggest that neither domains should be considered in isolation, and supports the rationale to combine biomechanical knowledge with psychologically informed principles throughout the assessment, treatment planning, and implementation phases of pain management. This appears particularly relevant for those individuals who exhibit higher fear of pain/injury and avoidance behaviour.

### Limitations

The results of this study should be discussed with consideration of several methodological limitations. In relation to the participant profiles, measures related to kinesiophobia deserve discussion. The TSK scores (36(7), range 23–49) were obtained from participants (n = 19) who were predominantly not seeking treatment for their LBP. If TSK values from this study (Mean = 36.3 (7.0)) are compared with TSK measures from other larger LBP studies [29], [31]–[33], it is evident that our population, while perhaps more generalizable in terms of a more typical LBP population, does not represent a highly kinesiophobic or fear avoidance presentation. For example, in the Leeuw et al. study [29] participants were excluded if they held TSK scores <34, whereas in this study only 9 of the 14 participants had a TSK score >34. Furthermore, the FABQ median split values used in this study (FABQ-W = 12, FABQ-PA = 11.5) are well below proposed elevated cut-off values (FABQ-W >34, FABQ-PA >15) used in previous studies [34] to identify individuals with high fear-avoidance beliefs. A further issue is that the participant sample size for this initial exploratory study (n = 19) may lack sensitivity to detect smaller effects. A follow-up study with a larger sample size is required to apply more vigorous statistics (i.e., multiple linear regression analysis, Bonferroni correction) and draw more robust conclusions related to this preliminary, exploratory finding. This is part of ongoing research.

### Future Directions

Results imply that trunk stiffness and kinesiophobia might serve as important moderators and/or mediators of persistent and recurrent LBP. This provides prioritization for future multi-system, biopsychosocial, and applied physiology investigative models to determine if pain management interventions aimed at targeting trunk mechanical properties and kinesiophobia reduce persistence or recurrence of LBP. Various movement based, cognitive and behavioural intervention strategies are worthy of investigation. The relationship between trunk mechanical properties and other pain psychology models (acceptance and commitment, misdirected problem solving, self-efficacy, and stress-diathesis) [35] and their accompanying psychological processes (i.e., cognitive flexibility in beliefs, attempts to solve problem, beliefs about the controllability of pain and coping skills, stress and anxiety) were not investigated. Likewise, other important psychological processes (i.e., attention and emotion regulation, distortion, expectations, helplessness, locus of control, stop rules, overt behaviour) and social-cultural-religious-environmental factors (i.e., spouse/co-worker/supervisor/spiritual support, job control, effort-reward imbalance, over-commitment) were not addressed and are important considerations.

Trunk stiffness and damping are one aspect of motor control, and other measurable dimensions, such as movement variability and movement-based subgroups could provide further context regarding the participant heterogeneity. The motor control task involved in this study is a measure of the participant’s automatic postural response and requires only slight forward or backward trunk movements, which may not reflect more planned and functional movement tasks that could potentially be more ‘fear inducing’ to the participant (i.e., forward bending, rotation of trunk or lifting). There has been a recent call in the literature to prioritize research aimed at providing a better understanding of the mechanisms by which yellow flags can affect the development of persistent pain and disability [36]. Physiology based studies, which examine both the motor control and psychological systems are ideally suited to serve this role and are part of ongoing research.

### Conclusion

The data suggest fear of movement (as measured by the TSK) relates, at least weakly, to trunk mechanical properties, which are considered to be an important component of the biological presentation of people with LBP. These findings lend further support to the necessity to recognise the interaction between biomechanical and psychological aspects of LBP rather than their consideration in isolation. It is possible that other psychosocial dimensions or pain psychology models may be related to biomechanical features (i.e., trunk mechanical properties, muscle activity, and movement patterns) in specific subgroups of LBP. Further integration of other potentially modifiable systems and more thorough investigation of the components within various pain psychology models hold promise in provision of a broader understanding of this multidimensional disorder.
